# Relative Deprivation and Suicidal Ideation Among Chinese College Students: The Mediating Role of Psychological Pain and the Moderating Role of Social Support

**DOI:** 10.3390/bs16101836

**Published:** 2026-10-08

**Authors:** Cui Lyu, Jing Lin, Kai Zhang, Shuyan Wang, Yanan Hu, Jia-Ming Wei, Bin Chang

**Affiliations:** 1 Department of Students’ Affair, Huaibei Normal University, Huaibei 235000, China; 2 Faculty of Psychology, Southwest University, Chongqing 400715, China; 3 College of Education, Huaibei Normal University, Huaibei 235000, China; 4 Quanzhou Academy of Cultural Heritage, Quanzhou Normal University, Quanzhou 362000, China

**Keywords:** relative deprivation, suicidal ideation, psychological pain, social support, college students

## Abstract

Suicidal ideation remains a longstanding and critical issue in mental health research due to its serious consequences for individuals’ lives and well-being. Although relative deprivation has been identified as an important risk factor for suicidal ideation, the mechanisms underlying this association remain poorly understood. Drawing on multiple theoretical perspectives, including the Strain Theory of suicide and the Stress-Buffering Model, this study examined a moderated mediation model linking relative deprivation to suicidal ideation, with psychological pain as the mediator and social support as the moderator. A cross-sectional survey was conducted among 630 college students. Participants completed self-report measures of relative deprivation, psychological pain, perceived social support, and suicidal ideation. The findings revealed that relative deprivation was positively associated with suicidal ideation and that psychological pain mediated this association (*β* = 0.066, 95% CI = [0.028, 0.121]). Moreover, social support significantly moderated both stages of the mediation process, weakening the positive effect of relative deprivation on psychological pain and the positive effect of psychological pain on suicidal ideation (*β* = −0.130, 95% CI = [−0.190, −0.070]; *β* = −0.089, 95% CI = [−0.150, −0.028]). Overall, the findings suggest that relative deprivation contributes to suicidal ideation through psychological pain and that social support serves as an important boundary condition in this process. These findings extend existing theoretical frameworks and offer practical implications for suicide prevention among college students.

## 1. Introduction

Suicidal ideation, defined as thoughts or considerations about ending one’s life, is widely recognized as a key precursor to suicidal behavior and an essential stage in the suicidal process (Hemming et al., 2019; Yosep et al., 2026; M. Zhao et al., 2025). Suicide remains a major public health concern worldwide and is currently the third leading cause of death among young people (World Health Organization, 2024). In China, it ranks as the second leading cause of non-disease-related death among youth (National Health Commission, 2024). College students may be particularly susceptible to suicidal ideation. During emerging adulthood, individuals undergo a developmental transition characterized by increasing autonomy and responsibility. At the same time, they encounter a range of challenges related to identity exploration, social relationships, academic performance, and future employment. Exposure to these concurrent stressors may heighten the likelihood of experiencing suicidal thoughts (Liu et al., 2025; Wang & Zhang, 2026; C. Wei et al., 2022; M. Zhao et al., 2025). The prevalence of suicidal ideation among Chinese college students underscores the urgency of this issue. A large-scale survey (*n* = 6836) found that approximately 18% of Chinese college students reported suicidal ideation (Wu et al., 2021). Consistent with this finding, a meta-analysis of 95,799 participants estimated the prevalence of suicidal ideation at 10.7% in this population (Tan et al., 2022). Given its high prevalence and substantial public health implications, suicidal ideation among Chinese college students warrants further investigation. Examining the psychological mechanisms underlying suicidal ideation from an integrative perspective may advance theoretical understanding of suicide-related processes and identify potential targets for intervention. Such knowledge may also inform evidence-based prevention strategies aimed at the early detection of suicidal risk and the reduction in suicide-related outcomes among college students.

### 1.1. Relative Deprivation and Suicidal Ideation

According to the Strain Theory of suicide (Zhang, 2019), relative deprivation represents an important risk factor contributing to suicidal tendencies. Relative deprivation is the judgment that one is worse off compared to some standard, accompanied by feelings of anger and resentment (Kuo et al., 2024; Osborne et al., 2012; Smith et al., 2012; Walker & Pettigrew, 1984; Walker & Smith, 2002). Empirical evidence from China and South Korea suggests that individuals who perceive greater relative deprivation tend to report higher levels of suicidal ideation (Pak & Choung, 2020; Zhang & Tao, 2013). According to Relative Deprivation Theory, perceiving oneself as relatively deprived may generate elevated psychological strain (Smith et al., 2012; Stamoulos & Prodromitis, 2026; Waldren & Callan, 2025; Walker & Pettigrew, 1984). Consistently, the Strain Theory of suicide proposes that relative deprivation may increase suicidal ideation by intensifying psychological strain (Zhang, 2019; Zhang & Tao, 2013). Therefore, psychological strain may serve as a mediating mechanism through which relative deprivation is associated with suicidal ideation.

### 1.2. The Mediating Role of Psychological Pain

Psychological pain represents a form of psychological strain (Zhang, 2019) and refers to an extreme, aversive emotional state that is chronic and intolerable (Baryshnikov & Isometsä, 2022; Shneidman, 1993; Troister & Holden, 2013). According to the Three-Step Theory (Klonsky et al., 2021), psychological pain is regarded as a core risk factor for suicidal thoughts and behaviors. When individuals experience severe psychological pain, they may consider suicide as a means of escaping from this intolerable state. In other words, psychological pain plays a central role in the development of suicidal ideation, as suicide may be perceived as a potential solution for terminating overwhelming psychological suffering (Klonsky et al., 2021; Li et al., 2014; Shneidman, 1993). Consistent with this theoretical perspective, empirical studies have shown that higher levels of psychological pain are associated with greater suicidal ideation (Ducasse et al., 2018; Landi et al., 2023; Liang et al., 2025).

Meanwhile, relative deprivation, as a chronic social stressor, has been consistently associated with psychological problems (Kuo et al., 2024; Smith et al., 2012). According to Relative Deprivation Theory (Smith et al., 2012; Walker & Pettigrew, 1984), perceived relative deprivation occurs when individuals believe that they have been denied deserved resources, opportunities, or outcomes, resulting in a discrepancy between their actual circumstances and their expected conditions. Such discrepancies may generate negative emotional experiences, including resentment, frustration, disappointment, dissatisfaction, and distress, thereby increasing vulnerability to psychological problems (Fan et al., 2025; Kalinowski & Rössler, 2025; Stamoulos & Prodromitis, 2026; Y. Xia & Ma, 2020). Importantly, the psychological consequences of relative deprivation closely resemble the characteristics of psychological pain, which is a complex negative emotional experience involving resentment, helplessness, and feelings of loss (Jin et al., 2024; Troister & Holden, 2013). Furthermore, unmet psychological needs represent a critical antecedent of psychological pain (Baryshnikov & Isometsä, 2022; Shneidman, 1993). Relative deprivation reflects such unmet needs, as individuals perceive a discrepancy between what they believe they deserve and what they actually obtain (Smith et al., 2012; Walker & Pettigrew, 1984; Zhang, 2019). Therefore, relative deprivation may increase suicidal ideation indirectly by eliciting psychological pain. Taken together, we propose the following hypothesis:

**H1.**  *Psychological pain mediates the association between relative deprivation and suicidal ideation.*

### 1.3. The Moderating Role of Social Support

Although relative deprivation and psychological pain represent significant risk factors for suicidal ideation among college students, their adverse effects may be attenuated by protective factors (Klonsky et al., 2021; Zhang, 2019). Social support has been widely recognized as a crucial protective factor that protects individuals against the harmful effects of psychosocial adversity and promotes psychological well-being. Social support refers to individuals’ perception of psychological, behavioral, and material resources (available or acted upon) in their social network (Uchino et al., 2012; Yeo et al., 2025).

According to the Stress-Buffering Model of social support (Cohen & Wills, 1985), social support can buffer the association between risk factors (e.g., stress) and psychological problems. The availability of social support enables individuals to obtain cognitive, emotional, informational, and material resources, thereby fostering reappraisal of stressors and enhancing coping flexibility, which ultimately attenuates the negative influence of risk factors on mental health (Cohen & Wills, 1985; Coyne, 1991). Empirical evidence has demonstrated that when individuals perceive higher levels of social support, the associations between risk factors (e.g., psychological strain) and psychological problems (e.g., suicide-related outcomes) become weaker (Lew et al., 2019; Perret et al., 2021; D. Zhao & Zhou, 2024).

Based on these theoretical perspectives and empirical findings, we propose that social support may buffer the adverse effects of relative deprivation, which represents a social stressor (Smith et al., 2012; Zhang, 2019), on psychological outcomes, including psychological pain and suicidal ideation. Therefore, we propose the following hypotheses:

**H2.**  *Social support weakens the positive association between relative deprivation and psychological pain.*

**H3.**  *Social support weakens the positive association between relative deprivation and suicidal ideation.*

Furthermore, the buffering effect of social support operates at multiple levels. Specifically, social support may not only attenuate the association between relative deprivation and psychological pain but also prevent the escalation of psychological pain into more severe outcomes, such as suicidal ideation. According to the Three-Step Theory of suicide (Klonsky et al., 2021), although psychological pain represents a critical factor contributing to suicidal desire, it only leads to intense suicidal desire when individuals experience a severe lack of connectedness (Pachkowski et al., 2021; Yang et al., 2018). Connectedness refers to an individual’s connection with people, activities, or other aspects of life that provide a sense of meaning and purpose, such as healthy interpersonal relationships (e.g., harmonious family relationships and intimate partnerships) and meaningful work (Klonsky et al., 2021; Klonsky & May, 2015). Social support represents a primary interpersonal manifestation of connectedness. Individuals with higher levels of social support are more likely to maintain close and positive relationships with others and experience greater meaning and fulfillment in life (Preston & Rew, 2021; Teo et al., 2020; Wickramaratne et al., 2022), thereby reducing their vulnerability to suicidal outcomes (Klonsky et al., 2021; Yang et al., 2018). Thus, social support may buffer the detrimental effect of psychological pain on suicidal ideation. Accordingly, we proposed the following hypothesis:

**H4.**  *Social support weakens the positive association between psychological pain and suicidal ideation.*

### 1.4. The Current Study

**Figure 1:**
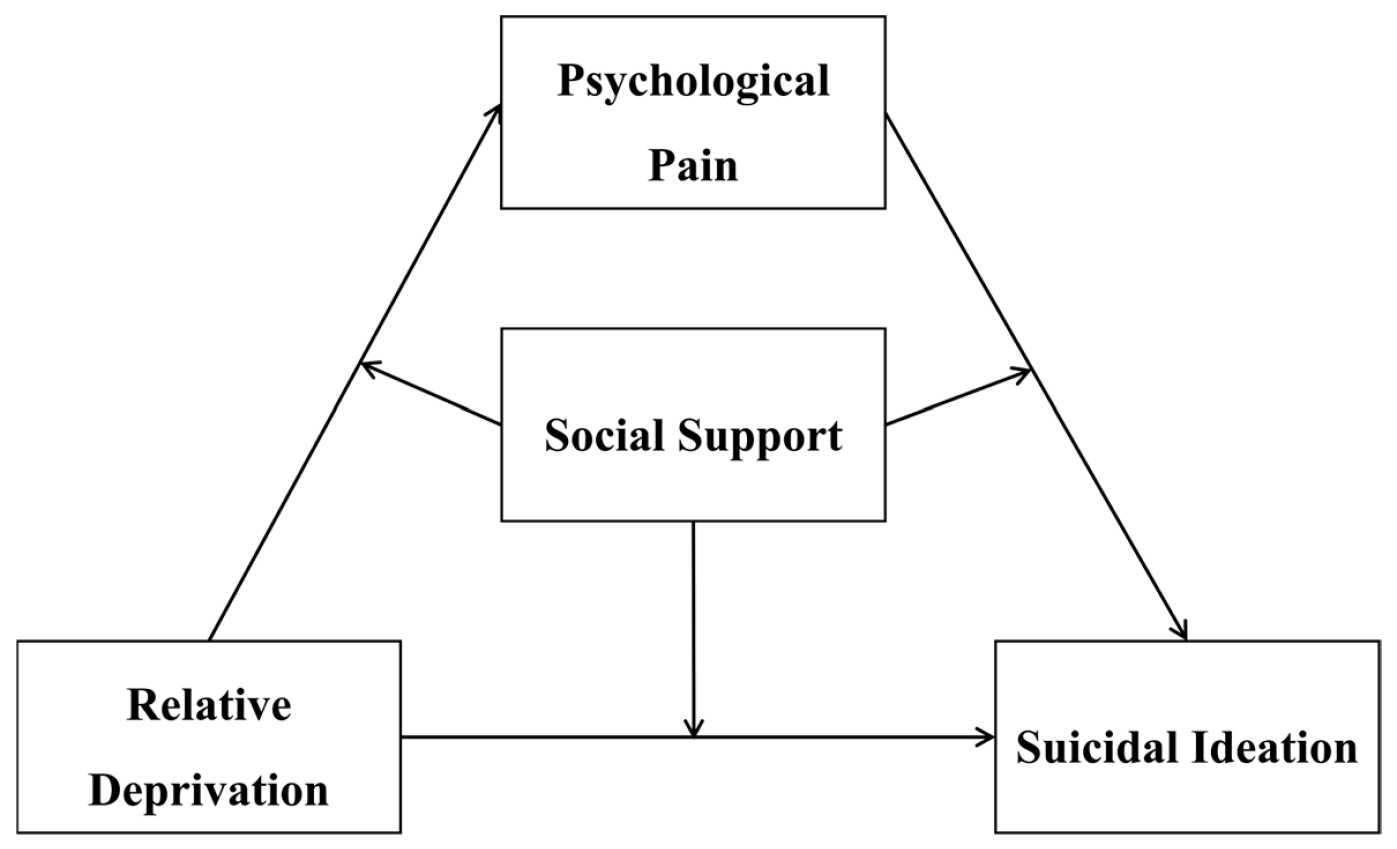
Proposed research model.

Previous research has established associations between relative deprivation and suicidal ideation (Pak & Choung, 2020; Zhang & Tao, 2013) and between psychological pain and suicidal ideation (Landi et al., 2023; Liang et al., 2025). Research has also indicated that social support is associated with weaker links between psychosocial risk factors and adverse psychological or suicide-related outcomes (Lew et al., 2019; Perret et al., 2021; D. Zhao & Zhou, 2024). However, these lines of research have largely examined the roles of psychological pain and social support separately. Less is known about whether psychological pain statistically accounts for the association between relative deprivation and suicidal ideation and whether social support moderates this indirect association at different stages. To address this gap, the present study integrated these variables into a moderated mediation model (see Figure 1). Specifically, we examined whether psychological pain mediated the association between relative deprivation and suicidal ideation and whether social support moderated the associations between relative deprivation and psychological pain, between psychological pain and suicidal ideation, and between relative deprivation and suicidal ideation.

The present study is expected to make both theoretical and practical contributions. Theoretically, it integrates the roles of psychological pain and social support within a single framework, thereby providing a more comprehensive account of the associations among relative deprivation, psychological pain, social support, and suicidal ideation. Practically, examining these associations may help identify potential targets for the prevention of suicidal ideation among college students, particularly psychological pain and social support.

## 2. Materials and Methods

### 2.1. Participants

This study employed a convenience sampling method, distributing questionnaires at the class level to collect data. The participants were full-time undergraduates enrolled at a university in Anhui Province, China. All participants had normal literacy skills, reported no history of psychological disorders, and had not received any psychological treatment within the six months prior to the survey. This was screened using two items: ① Have you been diagnosed with a psychological disorder (e.g., depression, anxiety, etc.) within the past six months? ② Have you received any psychological treatment (including counseling, pharmacotherapy, etc.) within the past six months? This exclusion criterion was applied to minimize the potential confounding effects of recent clinical or treatment-related experiences on the psychological variables examined in the present study. Such experiences may be associated with psychological pain, suicidal ideation, and perceived social support, making it more difficult to distinguish the associations of interest from those potentially related to recent psychological difficulties or treatment. All participants voluntarily took part in the investigation after providing informed consent and were free to ask the researchers for clarification regarding any questions encountered during questionnaire completion. The survey was conducted anonymously and did not involve any disclosure of private information. Participants were assured that the questionnaire data would be used solely for academic research and would be kept strictly confidential. This study complied with the requirements of the Declaration of Helsinki and was approved by the Ethics Committee of Huaibei Normal University (Approval No. HBNU-20260306).

A priori power analysis was conducted with G*Power 3.1 (Faul et al., 2009) to generate a conservative estimate of the required sample size, following the parameters reported in the literature (Lyu et al., 2026; J. Wei & Xia, 2023). The present study includes 11 predictors (gender, age, grade, urban or rural source, only child or not, major, relative deprivation, psychological pain, social support, relative deprivation × social support, psychological pain × social support). The analysis used the “*F* tests” test family and the “Linear multiple regression: Fixed model, *R*^2^ deviation from zero” statistical test. The analysis assumed a small effect size (*f*^2^ = 0.05), an *α* level of 0.05, and a desired statistical power of 0.95 (1 − *β*). These specifications yielded a minimum sample size of 513 participants.

A total of 663 questionnaire responses were collected. During the data cleaning phase, we screened the questionnaires for validity based on the following four pre-defined criteria: (1) missing responses (i.e., any item missing from any scale); (2) extreme response patterns (i.e., selecting the same option for all items); (3) formulaic responses (e.g., obvious repetitive response sequences); and (4) a score of ≥4 on the disguise dimension. These criteria were independently assessed by two researchers. After excluding questionnaires that met any of these criteria, 630 valid responses were retained, yielding a valid retention rate of 95.02%, which met the requirement of a priori power analysis. The participants ranged in age from 17 to 25 years, with a mean age of 19.75 years (*SD* = 1.11), including 161 males and 469 females. Among them, 264 were majoring in humanities/social sciences, 268 in science/engineering, and 98 in arts; 174 participants were from urban areas and 456 from rural areas; 151 were only children and 479 were not; and in terms of grade level, 132 were freshmen, 315 were sophomores, 174 were juniors, and 9 were seniors.

### 2.2. Measures

#### 2.2.1. Relative Deprivation Questionnaire

The Relative Deprivation Questionnaire, developed by Ma (2012) for Chinese college students, was employed. The scale consists of four items rated on a 6-point Likert scale (1 = strongly disagree, 6 = strongly agree). Scale scores were calculated as the mean of all items, with higher scores indicating greater perceived relative deprivation. In this study, the Cronbach’s *α* coefficient for the questionnaire was 0.759. The structural validity of the questionnaire in the present study was examined using confirmatory factor analysis (CFA). The results were as follows: *χ*^2^ = 1.008, *df* = 2, RMSEA = 0.000, CFI = 1.000, TLI = 1.004, and SRMR = 0.008.

#### 2.2.2. Self-Rating Idea of Suicide Scale

Suicidal ideation was assessed using the Self-Rating Idea of Suicide Scale developed by C. Y. Xia et al. (2012) for Chinese college students. The scale consists of 26 items rated using a dichotomous response format (0 = not consistent with the participant’s situation, 1 = consistent) and includes four dimensions: hopelessness, optimism, sleep, and disguise. In the present study, suicidal ideation was indexed by averaging the scores of all items from the hopelessness, optimism, and sleep dimensions. The disguise dimension functioned as a validity scale to assess response reliability. A cutoff score of ≥4 indicated careless or insincere responding, rendering the corresponding data invalid. Items 1, 5, 6, 7, 9, 10, 13, 15, 21, and 25 were reverse-scored. The scale demonstrated acceptable internal consistency in the present sample, with a Cronbach’s α of 0.733. The structural validity of the questionnaire in the present study was examined using CFA. The results were as follows: *χ*^2^ = 915.898, *df* =293, RMSEA = 0.058, CFI = 0.808, TLI = 0.787, SRMR = 0.063.

#### 2.2.3. The Psychache Scale

Psychological pain was assessed using the Chinese version of the Psychache Scale developed by Holden et al. (2001) and adapted for Chinese college students by Yang et al. (2018). The scale consists of a unidimensional structure with 13 items, rated on a 5-point Likert scale ranging from 1 (“never”) to 5 (“almost always”). The mean score across all items was calculated, with higher scores indicating greater levels of psychological pain. In the present study, the scale demonstrated excellent internal consistency, with a Cronbach’s α of 0.954. The structural validity of the questionnaire in the present study was examined using CFA. The results were as follows: *χ*^2^ = 541.479, *df* = 65, RMSEA = 0.108, CFI = 0.928, TLI = 0.913, SRMR = 0.042.

#### 2.2.4. Perceived Social Support Scale

The Perceived Social Support Scale, developed by Zimet et al. (1988), was employed. The scale comprises 12 items rated on a 7-point Likert scale (1 = strongly disagree, 7 = strongly agree). Scale scores were computed as the mean of all items, with higher scores reflecting higher levels of perceived social support. Previous studies have demonstrated acceptable reliability and validity of this scale in Chinese college student samples (Cheng et al., 2022; G. Zhao et al., 2022). In the current study, Cronbach’s α coefficient for the scale was 0.902. The structural validity of the questionnaire in the present study was examined using CFA. The results were as follows: *χ*^2^ = 262.555, *df* = 51, RMSEA = 0.081, CFI = 0.946, TLI = 0.930, SRMR = 0.041.

### 2.3. Data Analysis

All statistical analyses were performed using SPSS 27.0. First, internal consistency reliability of the scales was assessed, and descriptive statistics, correlation analyses, and Harman’s single-factor test (to examine common method bias) were conducted. Subsequently, the proposed moderated mediation model was tested using the PROCESS macro (version 4.0) for SPSS. Specifically, Model 4 was employed to examine the mediating effect of psychological pain on the relationship between relative deprivation and suicidal ideation, and then Model 59 was used to test the moderating role of social support on both the direct and indirect paths. Continuous variables were standardized before constructing the interaction terms. In all analyses, participants’ gender, age, grade, urban/rural origin, only-child status, and academic major (the variable was dummy-coded) were included as control variables. Parameter estimation was performed using the bias-corrected bootstrapping method with 5000 resamples, and a 95% confidence interval (95% CI) that did not include 0 was considered statistically significant. Before conducting the regression analyses, we assessed the assumptions of linearity, homoscedasticity, normality of residuals, influential observations, and multicollinearity. The results indicated that these assumptions were generally satisfied.

## 3. Results

### 3.1. Common Method Bias Test

Given that all variables were assessed using self-report measures, Harman’s single-factor test (Podsakoff et al., 2003) was conducted to examine the potential influence of common method bias. The results revealed 11 factors with eigenvalues greater than 1, with the first factor accounting for 21.988% of the total variance, which was below the recommended threshold of 40%. Thus, Harman’s single-factor test did not indicate a dominant single-factor structure.

### 3.2. Descriptive Statistics and Correlation Analysis

Descriptive statistics and Pearson correlation coefficients among the study variables are presented in Table 1. Relative deprivation was significantly positively correlated with suicidal ideation (*r* = 0.243, *p* < 0.001) and psychological pain (*r* = 0.238, *p* < 0.001), and significantly negatively correlated with social support (*r* = −0.274, *p* < 0.001). Suicidal ideation was significantly positively correlated with psychological pain (*r* = 0.353, *p* < 0.001) and significantly negatively correlated with social support (*r* = −0.361, *p* < 0.001). Psychological pain was significantly negatively correlated with social support (*r* = −0.306, *p* < 0.001).

### 3.3. Testing of the Mediating Role of Psychological Pain

The mediation analysis was conducted using Model 4 of the PROCESS macro (version 4.0) for SPSS (Hayes, 2013). Gender (0 = male, 1 = female), age, grade (0 = freshmen, 1 = sophomores, 2 = juniors, 3 = seniors), urban/rural origin (0 = rural, 1 = urban), only-child status (0 = yes, 1 = no), and academic major were included as control variables. The results of the mediation analysis are presented in Table 2. Specifically, the direct effect of relative deprivation on suicidal ideation was significant (*β* = 0.165, *p* < 0.001). Relative deprivation was significantly positively associated with psychological pain (*β* = 0.219, *p* < 0.001), and psychological pain was significantly positively associated with suicidal ideation (*β* = 0.303, *p* < 0.001). Bootstrap analysis indicated that the indirect effect of relative deprivation on suicidal ideation through psychological pain was 0.066, with a 95% bootstrap confidence interval that did not include 0. The indirect effect accounted for 28.57% of the total effect (0.066/0.231). Taken together, these findings demonstrated a significant mediating effect of psychological pain, supporting Hypothesis 1.

### 3.4. Testing the Moderating Role of Social Support in the Mediation Model

The moderating role of social support in the mediation model was examined using Model 59 of the PROCESS macro for SPSS (Hayes, 2013). The results are presented in Table 3. After including social support as a moderator, the indirect effect through psychological pain remained significant. Specifically, the direct effect of relative deprivation on suicidal ideation was significant (*β* = 0.108, *p* < 0.001). Relative deprivation was significantly positively associated with psychological pain (*β* = 0.157, *p* < 0.001), and psychological pain was significantly positively associated with suicidal ideation (*β* = 0.211, *p* < 0.001).

Furthermore, social support significantly moderated both the first and second stages of the mediation model. First, the interaction term between social support and relative deprivation significantly negatively predicted psychological pain (*β* = −0.130, *p* < 0.001), with the 95% bootstrap confidence interval excluding 0. Second, the interaction term between social support and psychological pain significantly negatively predicted suicidal ideation (*β* = −0.089, *p* < 0.01), with the 95% confidence interval excluding 0. However, the interaction term between social support and relative deprivation did not significantly predict suicidal ideation (*β* = 0.029, *p* = 0.435), indicating that social support did not significantly moderate the direct association between relative deprivation and suicidal ideation. The interaction between relative deprivation and social support explained an additional 2.4% of the variance in psychological pain (Δ*R*^2^ = 0.024, *F* = 17.966, *p* < 0.001), and the interaction between psychological pain and social support explained an additional 1% of the variance in suicidal ideation (Δ*R*^2^ = 0.01, *F* = 8.276, *p* < 0.001). In contrast, the interaction between relative deprivation and social support did not significantly predict suicidal ideation directly (Δ*R*^2^ = 0.001, *F* = 0.611, *p* > 0.05).

**Figure 2:**
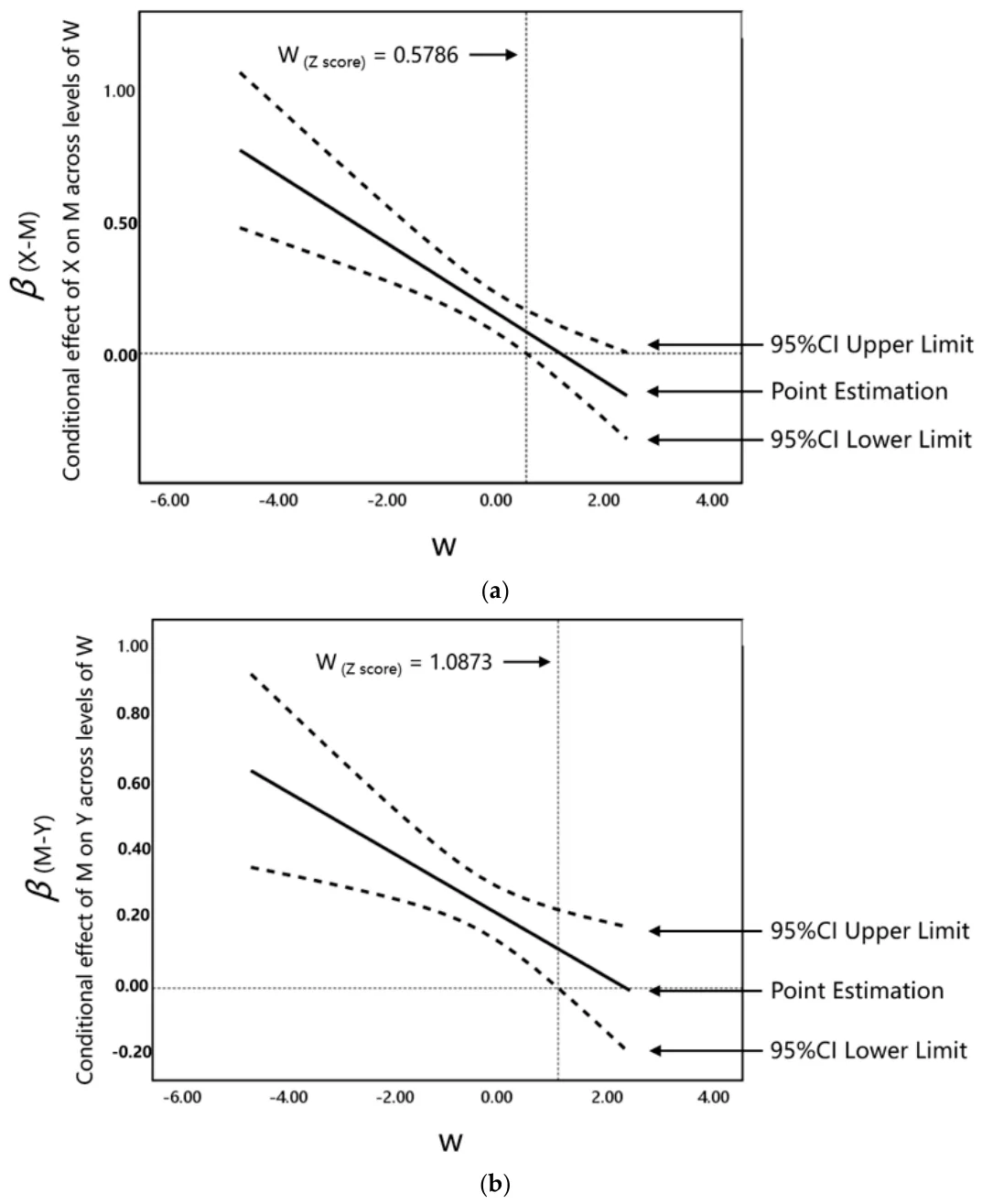
(**a**) Moderating effect of social support on the relationship between relative deprivation and psychological pain. (**b**) Moderating effect of social support on the relationship between psychological pain and suicidal ideation. Note: X = Relative Deprivation; M = Psychological Pain; Y = Suicidal Ideation; W = Social Support.

To further interpret the significant interaction effects, the Johnson-Neyman technique was applied to examine the conditional effects of relative deprivation on psychological pain and psychological pain on suicidal ideation. The results showed that the positive association between relative deprivation and psychological pain became significantly weaker as social support increased. Specifically, when social support exceeded 0.579 SD above the mean, the association between relative deprivation and psychological pain was no longer significant (see Figure 2a). Similarly, the positive association between psychological pain and suicidal ideation became significantly weaker as social support increased. When social support exceeded 1.087 SD above the mean, the association between psychological pain and suicidal ideation became non-significant (see Figure 2b). In other words, social support buffered the association between relative deprivation and psychological pain and weakened the positive association between psychological pain and suicidal ideation. Collectively, these findings supported Hypotheses 2 and 4, whereas Hypothesis 3 was not supported.

Moreover, to examine whether the indirect effect varied across levels of social support, we calculated the conditional indirect effects of relative deprivation on suicidal ideation through psychological pain at low (−1 SD), mean, and high (+1 SD) levels of social support (see Table 4). The conditional indirect effect was 0.300 (95% CI = [0.211, 0.389]) at low social support, 0.211(95% CI = [0.131, 0.290]) at mean social support, and 0.121 (95% CI = [0.012, 0.231]) at high social support. The indirect effect decreased as social support increased, indicating that the indirect association between relative deprivation and suicidal ideation through psychological pain was weaker at higher levels of social support.

## 4. Discussion

The present study sought to further elucidate the association between relative deprivation and suicidal ideation among Chinese college students by developing a moderated mediation model. Specifically, the findings demonstrated that psychological pain mediated the association between relative deprivation and suicidal ideation, whereas social support moderated both the association between relative deprivation and psychological pain and the association between psychological pain and suicidal ideation. The present study provides a more comprehensive understanding of how relative deprivation is related to suicidal ideation and offers important implications for suicide prevention and mental health promotion among college students.

### 4.1. Association Between Relative Deprivation and Suicidal Ideation

The present study demonstrated that relative deprivation was positively associated with suicidal ideation among college students, which is consistent with previous theoretical perspectives (Zhang, 2019) and empirical findings (Pak & Choung, 2020; Zhang & Tao, 2013). Relative deprivation reflects a negative cognitive evaluation whereby individuals perceive themselves as occupying an unfavorable position relative to others, which is often accompanied by feelings of resentment, unease, and sadness (Smith et al., 2012; Walker & Pettigrew, 1984). According to Relative Deprivation Theory and the Strain Theory of suicide, such psychological disadvantage may be associated with heightened psychological strain and greater vulnerability to suicidal ideation (Smith et al., 2012; Walker & Pettigrew, 1984; Zhang, 2019). Consistent with previous evidence from China and South Korea (Pak & Choung, 2020; Zhang & Tao, 2013), the present finding provides further empirical support for the positive association between relative deprivation and suicidal ideation among college students.

### 4.2. The Mediating Effect of Psychological Pain

The present study found that psychological pain mediated the association between relative deprivation and suicidal ideation among college students, providing support for Hypothesis 1. Specifically, greater perceived relative deprivation was associated with higher levels of psychological pain, which was also associated with higher levels of suicidal ideation.

Perceived relative deprivation reflects a sense of unmet entitlement, whereby individuals believe that they have not received what they deserve (Smith et al., 2012; Walker & Pettigrew, 1984). The discrepancy between perceived entitlement and actual outcomes may be associated with various negative emotional experiences, including resentment, disappointment, dissatisfaction, and sadness (Osborne et al., 2012; Smith et al., 2012; Walker & Pettigrew, 1984; Walker & Smith, 2002). These emotional experiences share important characteristics with psychological pain, which has been conceptualized as a complex of negative emotions and aversive psychological experiences (Jin et al., 2024; Troister & Holden, 2013). Thus, psychological pain may represent an important psychological manifestation of the negative emotional experiences associated with relative deprivation.

Psychological pain represents a central factor associated with suicidal ideation. Higher levels of psychological pain may be associated with greater suicidal ideation when psychological suffering is experienced as intense and persistent (Baryshnikov & Isometsä, 2022; Mee et al., 2006; Shneidman, 1993). Consistent with this perspective, empirical studies have shown that higher levels of psychological pain are associated with greater suicidal ideation (Ducasse et al., 2018; Landi et al., 2023; Liang et al., 2025).

Overall, the present finding that psychological pain statistically accounted for part of the association between relative deprivation and suicidal ideation is consistent with the Strain Theory of suicide (Zhang, 2019), which conceptualizes psychological pain as a form of strained mentality. This finding is also consistent with the Cubic Model (Shneidman & Farberow, 1957) and the Three-Step Theory (Klonsky et al., 2021), both of which highlight the relevance of psychological pain to suicidal ideation.

### 4.3. The Moderating Effect of Social Support

The present study found that social support buffered the association between relative deprivation and psychological pain. Specifically, compared with college students who perceived lower levels of social support, those who perceived higher levels of social support exhibited a weaker positive association between relative deprivation and psychological pain. Furthermore, social support also attenuated the association between psychological pain and suicidal ideation. As perceived social support increased, the positive association between psychological pain and suicidal ideation became progressively weaker. These findings provide support for Hypotheses 2 and 4.

The Risk-Protective Model of resilience (Zimmerman, 2013) proposes that protective factors, such as social support, can buffer individuals against the detrimental effects of stressors on mental health. Furthermore, the Stress-Buffering Model of social support (Cohen & Wills, 1985) suggests that social support may not only buffer the negative effects of stressful events on mental health but also attenuate the adverse consequences of psychological problems. For example, social support has been found to weaken the effects of negative repetitive thinking on loneliness (Fang et al., 2020) and on anxiety and depressive symptoms (Harrison et al., 2021). In the present study, social support attenuated both the association between relative deprivation and psychological pain and the association between psychological pain and suicidal ideation. These findings are consistent with the assumptions of the Risk-Protective Model and the Stress-Buffering Model of social support.

Higher levels of perceived social support indicate that individuals possess more supportive social networks, through which they can obtain various forms of assistance, including economic, emotional, and informational support (Cohen & Wills, 1985; Yeo et al., 2025). Therefore, for college students with higher levels of perceived social support, access to resources from their social networks may be associated with lower levels of psychological pain in the context of relative deprivation. Furthermore, social support also buffered the association between psychological pain and suicidal ideation. Consistent with the Three-Step Theory of suicide (Klonsky et al., 2021; Klonsky & May, 2015), stronger social connections may provide meaning and purpose, which may be particularly relevant when individuals experience substantial psychological pain. However, access to social support is influenced by structural and situational factors and is not distributed equally. Therefore, efforts to promote social support should not rely solely on students’ individual ability to form relationships but should also address inequalities in access to social support networks.

However, in the present study, social support did not significantly moderate the association between relative deprivation and suicidal ideation. Previous studies have also yielded inconsistent findings regarding the moderating role of social support in the association between stressful events and suicidal ideation. For example, Iwinski et al. (2026) found that social support buffered the effect of occupational stress on suicidal ideation among agricultural workers. In contrast, Panesar et al. (2021) reported that social support did not significantly moderate the association between stressful life events and suicidal ideation among individuals with psychiatric disorders. Similarly, Trujillo et al. (2017) found that social support from family, friends, and significant others failed to attenuate the association between discrimination and suicidal ideation among transgender sample.

In the present study, the direct pathway through which relative deprivation was associated with suicidal ideation may involve cognitive appraisal processes that were not fully explained by psychological pain. According to the Optimal Matching Theory of social support (Cutrona & Russell, 1990), social support is most effective when the type of support provided matches the specific demands of the stressor. However, the social support assessed in the present study primarily reflected emotional care and companionship from others, which may have limited relevance to the broader social and structural concerns embedded in relative deprivation, such as perceived inequality and unfairness. Although such interpersonal support may be relevant to psychological processes associated with relative deprivation, it may not be sufficient to address the broader social conditions underlying these perceptions. Therefore, the potential mismatch between the functions of social support and the broader demands associated with relative deprivation represents one possible explanation for why social support did not significantly moderate the direct association between relative deprivation and suicidal ideation. This possibility remains to be tested in future research.

### 4.4. Implications

Within the field of suicide research, the present findings provide empirical support for the Strain Theory and the Three-Step Theory. Previous research has separately documented the associations of relative deprivation and psychological pain with suicidal ideation, whereas the present study integrated these factors into a single model and showed that psychological pain statistically accounted for part of the association between relative deprivation and suicidal ideation. Moreover, this study extends the application of the Stress-Buffering Model of social support and Resilience Theory by showing that social support moderated the associations between relative deprivation and psychological pain and between psychological pain and suicidal ideation, but not the direct association between relative deprivation and suicidal ideation. These findings provide a more differentiated understanding of the roles of psychological pain and social support in the association between relative deprivation and suicidal ideation.

Furthermore, the present findings may have practical implications for suicide prevention and mental health promotion among college students. First, the findings highlight the potential relevance of students’ perceptions of inequality and relative deprivation, suggesting that universities and governmental agencies may consider creating fair and supportive environments that promote students’ sense of opportunity and future prospects. Second, the observed association between psychological pain and suicidal ideation highlights the importance of attending to students experiencing sustained and intolerable psychological pain, particularly in university mental health services. Third, the findings underscore the potential relevance of social support in the associations among relative deprivation, psychological pain, and suicidal ideation. Importantly, access to social support is shaped by structural and contextual factors and is not equally distributed. Therefore, efforts to promote social support should not rely solely on students’ individual capacity to build interpersonal relationships. Universities should integrate support resources such as mental health services, financial assistance, and informational support into routine institutional systems, thereby establishing institutionalized safety nets that ensure students can access support unconditionally without relying on personal networks. In addition, universities should develop proactive screening and early-warning mechanisms to identify at-risk students and provide support before they seek help These implications should be understood as potential areas for attention and future intervention research rather than as evidence that interventions targeting these factors would reduce suicidal ideation.

### 4.5. Limitations

Although the present study contributes to understanding suicidal ideation among college students and provides implications for its prevention, several limitations should be acknowledged.

First, given the cross-sectional design employed in this study, the temporal dynamics and causal relationships among variables could not be established. Although the proposed model specifies a particular ordering of the variables, reverse or reciprocal associations remain possible. Future research should employ longitudinal multi-wave designs and experimental approaches to further examine the proposed model, providing stronger evidence for its validity and identifying potential areas for refinement.

Second, all data in this study were obtained through participants’ self-reports, raising concerns about potential common method bias and social desirability bias. Although Harman’s single-factor test did not indicate a dominant single-factor structure, this result does not rule out the possibility of common method bias. Future studies may benefit from incorporating multiple assessment methods, including peer ratings, linguistic data, and ecological momentary assessment.

Third, the proposed model has only been examined in a sample of Chinese college students, which limits the generalizability of the findings. Given population differences in cultural backgrounds, economic conditions, and life developmental stages, the applicability of the present findings to other groups should be interpreted with caution. Future research should validate the proposed model in more diverse samples, such as adolescent samples and community adult samples, to further establish its external validity.

Fourth, the exclusion of students with previous psychological disorders or recent psychological treatment may have introduced selection bias and reduced the representation of students with elevated suicide risk. Future studies should include more diverse student populations to enhance the generalizability of the findings.

Fifth, the present study examined only the mechanism underlying the association between relative deprivation and suicidal ideation among college students. According to the Strain Theory of suicide (Zhang, 2019), in addition to relative deprivation, value strain, aspiration strain, and coping strain are also considered potential contributors to suicidal ideation. Therefore, future studies should systematically investigate the roles of these additional stressors in the association between stressors and suicidal ideation to obtain a more comprehensive understanding of suicide risk mechanisms.

Sixth, although the sample size met the requirement of the a priori power analysis, the power analysis was based on a conventional multiple-regression model and did not specifically estimate the statistical power for the interaction and indirect effects examined in the moderated mediation model. The use of convenience sampling may have introduced sampling bias and limited the representativeness of the sample. Thus, the findings should be interpreted with caution, and future studies should recruit more diverse samples using probability-based sampling methods to improve the generalizability of the findings.

Seventh, the present sample showed substantial demographic imbalance in gender and academic year. Female students accounted for 74.4% of the sample, whereas only nine participants were in their fourth year. Although gender and academic year were included as covariates, the imbalance may limit the generalizability of the findings across demographic groups and the extent to which the consistency of the observed associations across gender and academic-year groups can be evaluated. Future studies should recruit samples with more balanced distributions of gender and academic year and further examine whether the observed associations vary across these groups.

## 5. Conclusions

Consistent with previous research (Zhang, 2019; Zhang & Tao, 2013), the present study demonstrated that relative deprivation was associated with suicidal ideation among college students. Furthermore, psychological pain statistically mediated the association between relative deprivation and suicidal ideation. Importantly, perceived social support moderated both the association between relative deprivation and psychological pain and the association between psychological pain and suicidal ideation. Meanwhile, social support may function as a protective resource that moderates the associations between relative deprivation and psychological pain and between psychological pain and suicidal ideation among college students. Specifically, the indirect association between relative deprivation and suicidal ideation through psychological pain was weaker at higher levels of perceived social support.

Overall, the findings provide evidence for the associations among relative deprivation, psychological pain, social support, and suicidal ideation within the present model. Longitudinal research is needed to further examine the temporal and causal relationships among these variables.

## Figures and Tables

**Table 1:** Descriptive statistics and correlations between variables.

Variable	M ± SD	Kurtosis	Skewness	1	2	3	4
1.Relative Deprivation	2.720 ± 0.788	0.137	0.154	1			
2. Suicidal Ideation	0.257 ± 0.147	0.195	0.791	0.243 ***	1		
3. Psychological Pain	1.526 ± 0.552	8.883	2.335	0.238 ***	0.353 ***	1	
4. Social Support	4.952 ± 0.840	0.251	0.025	−0.274 ***	−0.361 ***	−0.306 ***	1

Note. *** = *p* < 0.001.

**Table 2:** Mediating effect of psychological pain between relative deprivation and suicidal ideation.

Path	Effect Size	*SE*	95% Confidence Interval
Direct Path: Relative Deprivation → Suicidal Ideation	0.165	0.038	0.091, 0.239
Indirect Path: Relative Deprivation → Psychological Pain → Suicidal ideation	0.066	0.024	0.028, 0.121
Total effect	0.231	0.039	0.156, 0.307

**Table 3:** Testing the proposed moderated mediation model.

Variable	Model 1 (Psychological Pain)			Model 2 (Suicidal Ideation)		
	*β*	*t*	95% CI	*β*	*t*	95% CI
Relative Deprivation	0.157	4.148 ***	0.082, 0.231	0.108	2.876 **	0.034, 0.181
Psychological Pain				0.211	5.236 ***	0.132, 0.290
Social Support	−0.259	−6.730 ***	−0.334, −0.183	−0.240	−6.157 ***	−0.316, −0.163
Relative Deprivation × Social Support	−0.130	−4.239 ***	−0.190, −0.070	0.029	0.782	−0.044, 0.102
Psychological Pain × Social Support				−0.089	−2.877 **	−0.150, −0.028
Gender	−0.410	−4.693 ***	−0.581, −0.238	0.043	0.496	−0.127, 0.214
Age	−0.027	−0.625	−0.110, 0.057	0.021	0.502	−0.061, 0.103
Major	−0.041	−0.769	−0.144, 0.063	0.025	0.478	−0.076, 0.126
Grade	0.223	3.329 ***	0.091, 0.355	0.075	1.133	−0.055, 0.205
Urban/Rural Origin	0.072	0.842	−0.097, 0.241	0.064	0.759	−0.101, 0.230
Only-child Status	−0.008	−0.087	−0.185, 0.170	0.109	1.227	−0.06, 0.283
*R*^2^ (*F*)	0.190 (16.129 ***)			0.230 (16.723 ***)		
Δ*R*^2^ (*F*)	0.024 ^a^ (17.966 ***)			0.001 ^a^ (0.611)		
				0.01 ^b^ (8.276 ***)		

Note. ** = *p* < 0.01; *** = *p* < 0.001; CI = Confidence Interval; ^a^ indicates the interaction term “Relative Deprivation × Social Support”; ^b^ indicates the interaction term “Psychological Pain × Social Support”.

**Table 4:** Conditional Indirect Effects of Relative Deprivation on Suicidal Ideation Through Psychological Pain at Different Levels of Social Support.

Social Support Level	Conditional Indirect Effect	*SE*	95% Confidence Interval
Low (−1 SD)	0.300	0.045	0.211, 0.389
Mean	0.211	0.040	0.131, 0.290
High (+1 SD)	0.121	0.056	0.012, 0.231

## Data Availability

The research data are available upon reasonable request from the primary corresponding author (J.-M.W.).
